# Evaluation of *Lactiplantibacillus plantarum* KAU007 against Low-Pathogenic Avian Influenza Virus (H9N2)

**DOI:** 10.3390/pathogens11111246

**Published:** 2022-10-27

**Authors:** Irfan A. Rather, Majid Rasool Kamli, Jamal S. M. Sabir, Sajad Ali

**Affiliations:** 1 Department of Biological Sciences, Faculty of Science, King Abdulaziz University, Jeddah 21589, Saudi Arabia; 2 Center of Excellence in Bionanoscience Research, King Abdulaziz University, Jeddah 21589, Saudi Arabia; 3 Department of Biotechnology, Yeungnam University, Gyeongsansi 38541, Gyeongsanbuk-do, Korea

**Keywords:** probiotics, pathogen, influenza, H9N2, KAU007, infection

## Abstract

Avian influenza A viruses (AIVs) pose a persistent threat to humans owing to their reassortment and antigenic drift properties. Among them is H9N2, a low-pathogenic avian influenza virus first discovered in the non-human host and later found infective to humans with huge pandemic potential. In recent years, antiviral resistance has become an increasing threat to public health. Additionally, vaccination against AIVs is becoming increasingly challenging with little success due to antigenic drift. This has resulted in a growing demand for products that can replace the presently in-use medications and the development of innovative antiviral therapies. In this study, we systematically investigate the antiviral potential of lactic acid bacteria against H9N2. Bacteria that produce lactic acid are commonly used in food processing. In addition, these bacteria are considered more affordable, effective, and safe “nutraceuticals” than other alternative medicines. We tested *Lactiplantibacillus plantarum* KAU007 against the low-pathogenic avian influenza virus (H9N2). As confirmed by the hemagglutination assay, KAU007 showed potent antiviral activity against H9N2 and vigorous antioxidant activity. The CFCS showed a dose-dependent reduction in the levels of IL-6 and IFN-γ. Thus, KAU007 might be considered a potential H9N2 target-based probiotic.

## 1. Introduction

Viral diseases with pandemic potential have significantly miffed the world population and economy for centuries. A recent study has shown that climate change will force new animal encounters that could drive the emergence of more newly viral diseases [1]. In humans, zoonotic transmission of viral pathogens has been a critical route for newly emerging viruses that have afflicted them for decades. Avian influenza A viruses (AIVs) have become a major threat to humans and livestock across the globe. Recent outbreaks due to newly emerged AIVs in poultry sectors have been the most devastating factors and human concern [2,3]. AIVs belong to the Orthomyxoviridae family and are enveloped pleomorphic with an eight-segmented single-stranded RNA genome [4]. Based on their hemagglutinin (HA) and neuraminidase (NA) antigenic properties, AIVs are grouped into diverse subtypes, viz., HA (H1-H18) and NA (N1-N11) [5]. Owing to their antigenic drift and shift features, the resurgence of AIVs may pose a future pandemic and zoonotic threat to any country, necessitating sporadic monitoring and developing of efficient antiviral therapy. Based on the pathotyping, they have been classified into high and low pathogenic AIVs and are becoming endemic to Asian and African regions. The fast and constant evolution of AIVs makes surveillance and control extremely difficult. Several entirely AIVs have broken the species barrier in recent years, causing human illness and even death. AIV subtypes such as H5N1, H6N1, H7N9, H9N2, H10N8, and H5N6 are widely circulating and posing a threat to humans across the globe [6]. Among AIVs, H9N2 has caused significant economic losses, especially when co-infected with other respiratory illnesses [7,8,9]. In 1966, the H9N2 virus was isolated from turkeys in the US state of Wisconsin and now, it is known that it can infect broad hosts such as chickens, quail, ducks, pigs, geese, and humans [10,11]. Phylogenetic analysis of H9N2 viruses has revealed several clades belonging to the G1-like lineage and the Y280-like lineages [12]. Based on pathotyping and molecular characterization, H9N2 subtype viruses are classified as low-pathogenicity avian viruses (LPAIV) with the ability to infect broad hosts such as chickens, turkeys, quail, ducks, pigs, geese, and humans. H9N2 has been a major threat to the poultry industry and humans, owing to its pandemic potential and high rates of zoonotic infections [13]. In 1998, China reported the first human infection with the H9N2 virus; since then, human infections have been documented in a number of nations, suggesting a severe concern in the future [11,14]. From 1998 and early 2021, 74 H9N2 human infections were reported, mostly in youngsters who had previously been exposed to poultry which further highlights its zoonotic potential [10,11,12,13,14]. Existing genetically diverse H9N2 viruses are considered a moderate pandemic risk due to their global dispersion, extensive host range, and reassortment capabilities [15]. Furthermore, it has been observed that the H9N2 strain of low pathogenic avian influenza virus (LPAIV) can easily undergo genetic reassortment and transfer internal gene segments to highly pathogenic avian influenza viruses (HPAIV) H5 and H7 [16,17]. For example, constant internal gene segment transfer of H9N2 leads to new human-infecting H5N1, H5N6, H7N9, and H10N8 influenza subtypes, highlighting the virus’s potential pandemic threat.

Vaccination is a cost-effective and efficient way to prevent and control virus outbreaks. Previous studies have revealed that immunizing chickens against AIVs such as HPAI H7N77 and H5N18 lower viral transmission rates [18]. As a result, inactivated vaccinations have been employed to prevent H9N2 epidemics in the poultry sector. Although several countries used inactivated vaccines to prevent disease, the virus continues to spread in vaccinated chicken farms, probably due to antigenic drift [19]. Although the antigenic drift may have been the primary cause of H9N2 virus failure, other factors such as insufficient vaccination coverage, inefficient vaccination use, or a low dose may also have contributed to vaccination failure [20]. As a result, finding new antiviral remedies for H9N2 is critical from both a human and a poultry perspective. In this regard, harnessing the potential of probiotic bacteria against AIVs such as H9N2 is a promising approach owing to its multiple beneficial traits to their hosts and protection. There have been reports of probiotics effectively treating viral infections of the intestine, respiratory system, and urogenital system [21]. Previous studies have shown that lactic acid bacteria (LAB) and their metabolites show solid antiviral activity against viral pathogens [22,23,24]. The suppression of virus replication appears to be one of the most essential antiviral mechanisms discovered so far using LAB [25]. However, LAB and their metabolites, such as bacteriocins, can also trigger the host immune response providing resistance to viral infections [22,23]. LAB strains have been effectively utilized to treat dental, gastrointestinal, and vaginal infections for many years, and some can even lower serum cholesterol [21]. Despite scientific and technological advances, the AIVs continue to threaten human health and the global economy. Hence, finding efficient antiviral agents and a better understanding of viral infections with the potential to cause pandemics can thus aid in preventing future pandemics. In this regard, LAB have a lot of potential for “virus warfare”, notably by reducing the use of toxic virucidal chemicals in several industries while simultaneously promoting growth. A recent study demonstrated that *L. plantarum* was an effective method of delivering antigens from pathogens to immunize against infections caused by these pathogens [26]. Therefore, this study aims to evaluate the antiviral effect of KAU007 isolated from camel milk against LPAIV.

## 2. Materials and Methods

### 2.1. Bacteria Culture and CFCS Preparation

*L. plantarum* KAU007 strain was previously isolated from camel milk [27]. To prepare the cell-free culture supernatant (CFCS), the isolate was cultured on de Man Rogosa (MRS) broth at 37 °C for 24 h, followed by centrifugation at 10,000 rpm at 4 °C for 10 min. The supernatant was transferred into a fresh sterile tube and syringe filtered using a 0.22 µm pore size syringe. The sterile CFCS of *L. plantarum* KAU007 was used in different concentrations viz., 2.5, 5, and 10 mg/mL.

### 2.2. Cell Culture, Cytotoxicity, and Antiviral Activity of CFCS of L. plantarum

The most frequently utilized cell line for the isolation and propagation of human influenza viruses (HIVs) is Madin–Darby canine kidney (MDCK) cells. In this study, the MDCK cell line was regularly cultivated in the lab using the same procedure as previously described [27,28,29,30]. Before evaluating the antiviral effect of *L. plantarum* CFCS on H9N2 grown in MDCK cell lines, we first examined the cytotoxic effect of *L. plantarum* CFCS on MDCK cells following the procedure [27]. H9N2 influenza virus (A/Korea/01/2009) was previously acquired from KCDA, Korea, and grown on MDCK cell lines for 72 h with 4% CO_2_ at 37 °C. CFCS of *L. plantarum* KAU007 was used to treat the H9N2 virus for 1 h at 37 °C in doses of 2.5, 5, and 10 mg/mL under 5% CO_2_. The CFCS-treated H9N2 was then injected into MDCK cell lines which were subsequently grown for 48 h at 37 °C in a humid environment with 5 percent CO_2_. After two days, the plates were checked for cytopathic effects (CPEs). 

### 2.3. Hemagglutination Assay

In this study, dual inoculation of H9N2 (10^6.5^ EID_50_/0.1 mL) and CFCS of *L. plantarum* (2.5, 5, and 10 mg/mL) were inoculated into 11 days embryonated eggs using microinjection syringe. The eggs were then incubated for 4 to 5 days in egg incubator with 70% humidity at 37 °C. Similarly, control eggs were inoculated with PBS. The survival rate of the embryonated eggs was then assessed. For hemagglutination test, the allantoic fluid from eggs chilled at 4 °C for 2 h was harvested into sterilize tubes. A two-fold dilution of treated samples was made in 50 µL using PBS in V shaped 96-well microplate. Finally, equal volume of 1% SPF chicken RBC was added to diluted samples and allowed to hemagglutination for 30 min [30].

### 2.4. Estimation of Cytokines and Antioxidant Activity after CFCS L. plantarum KAU007

In this work, the impact of CFCS on cytokine profile was assessed using three different CFCS dosages (2.5, 5, and 10 mg/mL). Briefly, cells were treated with CFCS, and IFN-γ and IL-6 levels were determined using quantitative sandwich ELISA kits (R&D Systems, Inc., Minneapolis, MN, USA), per the manufacturer’s recommendations. The plates were read at 450 nm. The results were given in mg of protein per plate.

The antioxidant activity of KAU007 was determined by estimating the nitrate scavenging ability [31], DPPH radical scavenging ability [32], and SOD-like activity by following the method previously described [32,33,34,35,36].

### 2.5. Statistical Analysis

Statistical significance was determined by comparing untreated control with treated groups using two-way ANOVA. The graphs were made using GraphPad Prism (v 9.4.1GraphPad Software, San Diego, CA, USA). Each experiment was conducted in triplicate, and data obtained were presented as means ± standard errors. A *p*-value ≥ 0.05 was considered significant.

## 3. Results and Discussion

*Lactobacillus plantarum* stains have been found to be a viable alternative for treating bacterial and viral infections owing to their potent antibacterial and antiviral properties [27,37]. In this study, we examined the antiviral activity of *L. plantarum* KAU007 (GenBank accession No. OM442911) against the low-pathogenic avian influenza virus (H9N2). H9N2 has been a major threat to the poultry industry and humans due to its pandemic potential and high rates of zoonotic infections. Therefore, developing a successful antiviral medication against H9N2 is crucial from a human and poultry perspective.

### 3.1. To Evaluate the Antiviral Effect of CFCS of L. plantarum against H9N2 in MDCK Cells

**Figure 1:**
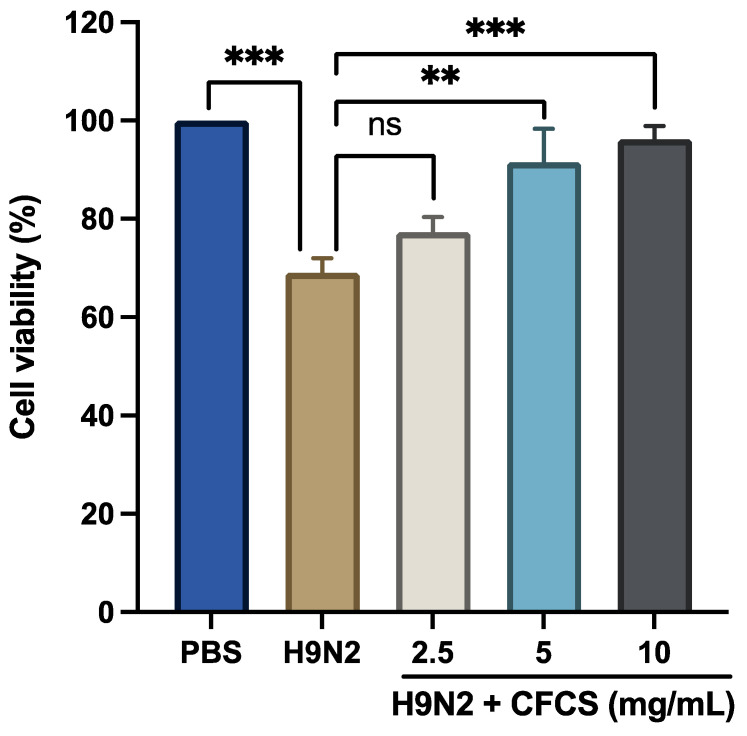
Cytopathic effect of the H9N2 virus. MTT assays were used to measure cell viability. Each experiment was performed three times, and the results are presented as a mean + standard error. To determine statistical significance, treatment groups were compared with control (PBS only) and H9N2 (no treatment). *p*-values: *** < 0.001, ** < 0.01. ns = non-significant.

Previously, we evaluated the cytotoxicity of CFCS by measuring the viability of MDCK cells that had been treated with different concentrations of CFCS using the MTT test [27]. The results showed no appreciable effects of different concentrations of CFCS on the MDCK cells after 24 h compared to the untreated control cells, demonstrating that CFCS of *L. plantarum* has no cytotoxic effects on mammalian cells. Previous studies have also revealed that CFCS has no effect on normal human cells but is highly cytotoxic to cancer cells [38,39]. Further, we systematically examined the antiviral activity of L. plantarum CFCS against H9N2. Firstly, we evaluate the H9N2-induced CPE in MDCK cells using a viral dose (10^6.5^ EID_50_/0.1 mL). Our results show a significant reduction of viable MDCK cells (*p* < 0.001) compared to control or uninfected MDCK cells. However, pretreatment of MDCK cells with *L. plantarum* CFCS (5 and 10 mg/mL) showed no H9N2-induced CPE after 72 h of post-viral infection compared to non-CFCS treated and infected cells (Figure 1). Interestingly, even after treating H9N2-infected cells with 2.5 mg/mL CFCS; (not significantly different from infected cells, *p* = 0.09); differences in the viability of the cells were still seen. These results are consistent with previous reports on the antiviral activity of CFCS of L. plantarum against various viral pathogens [29]. This adds to the growing body of evidence that has shown that probiotic bacteria and their metabolites are the most promising strategies for treating viral illnesses.

### 3.2. Evaluation of Antiviral Activity of L. plantarum CFCS against H9N2 Using Embryonated Eggs and Hemagglutination Assay

In this study, dual inoculation of H9N2 (10^6.5^ EID_50_/0.1 mL) and CFCS of L. plantarum (2.5, 5, and 10 mg/mL) were inoculated into embryonated eggs. The survival rate of the embryonated eggs was then assessed. Our findings showed that the most significant (*p* < 0.001) survival rate for embryonated eggs was enhanced by 10 mg/mL CFCS (Table 1). These findings add to the growing body of evidence supporting *L. plantarum’s* potential antiviral activity against H9N2 and suggest that it may represent a good candidate for antiviral treatment in the future. Alternatively, we investigated the hemagglutination assay (HA) to identify hemagglutinating substances in egg culture and amniotic fluid collected from embryonated eggs against H9N2. Our results showed that CFCS inhibited hemagglutination with all the three tested concentrations of 2.5 mg/mL, 5 mg/mL, and 10 mg/mL (Table 2). Previous studies have also revealed that *L. plantarum* AA09a can efficiently decrease viral infectivity [40].

### 3.3. Effect of L. plantarum CFCS on Proinflammatory Cytokines

**Figure 2:**
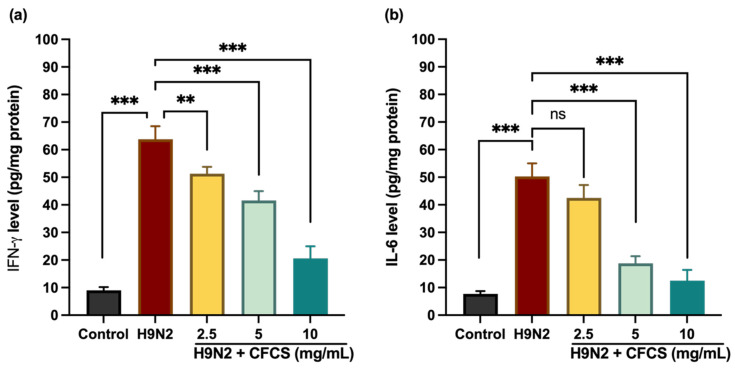
H9N2 virus-challenged mammalian kidney cells treated with CFCS show a reduction in interleukin levels. (**a**) IFN-γ and (**b**) IL-6. Results were presented as means + SE for the three experiments conducted in triplicate. *p*-values: ** < 0.01, *** < 0.001, ns = non-significant.

Virus infections trigger the expression of cytokines and chemokines as part of proinflammatory response. In this study, we examine the effect of *L. plantarum* CFCS on proinflammatory cytokines by monitoring the expression of two proinflammatory signature cytokines, IFN-γ and IL-6. Based on our findings, the expression levels of IFN-γ and IL-6 significantly decreased after CFCS treatment. In contrast, H9N2 infected cells showed dramatic increase in IFN-γ levels (62.80 ± 4.7 pg/mL) compared to uninfected or control cells (9 ± 1.2 pg/mL) (*p* < 0.001). Figure 2 shows that CFCS significantly reduced the level of IFN-γ in a dose-dependent manner at concentrations of 5 and 10 mg/mL, with *p* values of <0.001. However, there was no significant difference between H9N2-infected cells and CFCS (2.5 mg) treated cells in terms of IL-6 expression levels (*p* = 0.1). The expression levels of IL-6 were significantly higher in H9N2-infected cells than in non-infected cells. However, pretreatment of *L. plantarum* CFCS (5, and 10 mg/mL) significantly decreases the levels of IL-6 when compared to H9N2 infected cells (*p* < 0.001). Previous studies have shown that *L. plantarum* reduces proinflammatory cytokines such as TNF, IL-6, and IL-8 [40].

### 3.4. Effect of L. plantarum CFCS on Antioxidant System

**Figure 3:**
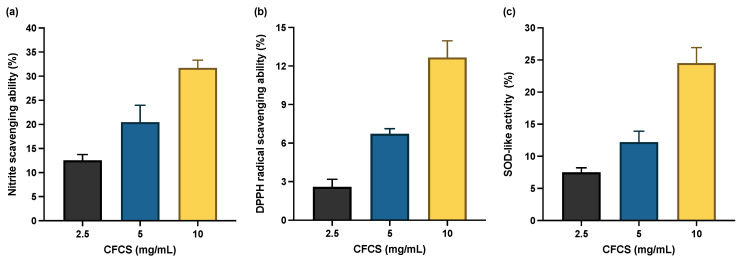
Antioxidant activity of CFCS isolated from *L. plantarum* KAU007 (**a**) nitrate radical scavenging activity of CFCS. (**b**) DPPH radical scavenging activity of CFCS. (**c**) Superoxide dismutase (SOD) such as activity of CFCS. Data are expressed as mean ± SD (*n* = 3).

Reactive nitrogen species (RNS) and reactive oxygen species (ROS) play a vital role in immunoreaction against microbial pathogens [41]. However, excess RNS/ROS production results in oxidative stress, which in turn causes DNA damage, lipid peroxidation, and protein oxidation [42]. *L. plantarum* bacteria strains induce antioxidant enzymatic activity, which protects cells from oxidative stress. In this study, we systematically investigate how *L. plantarum* KAU007 CFCS affects the activity of antioxidants, viz., nitrate radical scavenging activity, DPPH radical scavenging activity, and superoxide dismutase. According to our findings, CFCS (2.5, 5, and 10 mg/mL) significantly boosts the activity of all three antioxidants, but CFCS (10 mg/mL) exhibited the highest antioxidant activity as shown in Figure 3. The ability of *L. plantarum* KAU007 CFCS to scavenge free radicals was dosage-dependent, as shown in Figure 3. Previous investigations have also emphasized the significance of the *L. plantarum* strains in triggering antioxidant activity [43,44].

The use of probiotics in fermented foods and dairy products has a long evolutionary history of benefiting the host [34,36,45,46,47,48,49,50,51]. As dietary supplements, probiotics have become increasingly popular in recent decades. The effectiveness of probiotics has also been confirmed in thousands of scientific studies, demonstrating their effectiveness against various ailments, including bacterial, viral, and fungal infections [22,27,28,29,30,40,52,53,54,55,56,57]. Recent years have seen an increase in the popularity of LAB because of its antibacterial and probiotic properties. A growing body of research suggests that it may represent a promising alternative to synthetic drugs [58,59,60,61,62,63,64,65,66].

This study tested the effectiveness of camel milk isolate, *L. plantarum* KAU007 against the LPIV virus. In higher concentrations, KAU007 CFCS is more effective at nearly eradicating the virus. The isolated CFCS from KAU007 showed no cytotoxicity to MDCK cells [27]. Based on these results, the KAU007 could be considered a safe candidate that may not cause host cell damage. The in vitro antiviral activity assay conducted on *L. plantarum* KAU007 against the LPAI virus shows high antiviral activity. To combat LPAI, the most effective dose of CFCS was 10 mg/mL from KAU007.

We observed similar results in SPF embryonated eggs, with maximum antiviral activity at a concentration of 10 mg/mL of CFCS. Therefore, the study underpins that *L. plantarum* KAU007 is an anti-influenza prophylactic probiotic found in camel milk. Further, the results reinforce the study of the mechanism of action of KAU007 against LPAI, as well as the mechanisms of action against other influenza strains, in order to develop effective therapeutic candidates to prevent influenza virus-induced respiratory disease.

## 4. Conclusions

Using probiotic antagonist microbes and their metabolic products could be a promising therapeutic strategy against viral infections. In this study, we investigate the role of *L. plantarum* KAU007 against H9N2 using different approaches. Our results revealed that *L. plantarum* KAU007 has potent antiviral activity against H9N2 and could be a promising candidate for future antiviral therapy. In the future, multi-omics and other high throughput immunotechnological approaches will be needed to identify the metabolites in *L. plantarum* KAU007 responsible for its antiviral capabilities, which will open up new possibilities for the mechanisms underlying its antiviral capabilities in antiviral development.

## Figures and Tables

**Table 1:** An evaluation of the effects of CFCS on the survival rate of embryonated eggs infected with H9N2.

Groups	Treatment	Survival/Total	Survival Percentage
Con	PBS	2/2	100%
H9N2	10^7.5^ EID_50_/0.1 mL	5/9	55%
H9N2 + KAU007—I	CFCS (2.5 mg/mL)	3/4	75%
H9N2 + KAU007—II	CFCS (5 mg/mL)	5/6	83%
H9N2 + KAU007—III	CFCS (10 mg/mL)	8/9	88.88%

Viral titer: 10^6.5^ EID_50_/0.1 mL; Con: PBS only.

**Table 2:** Inhibition of hemagglutination by CFCS of *Lactiplantibacillus plantarum* KAU007.

Treatment				
Hemagglutinating activity	Con	KAU007 CFCS (2.5 mg/mL)	KAU007 CFCS (5 mg/mL)	KAU007 CFCS (10 mg/mL)
	0	1:4	1:8	1:8

Viral titer: 10^6.5^ EID_50_/Suppp0.1 mL; Con: PBS only.

## Data Availability

The data generated are cited in the manuscript.
